# Biharmonic-Drive Tunable Josephson Diode

**DOI:** 10.1021/acs.nanolett.5c03922

**Published:** 2025-09-17

**Authors:** Laura Borgongino, Rubén Seoane Souto, Alessandro Paghi, Giulio Senesi, Katarzyna Skibinska, Lucia Sorba, Elisa Riccardi, Francesco Giazotto, Elia Strambini

**Affiliations:** † NEST, Istituto Nanoscienze-CNR and Scuola Normale Superiore, I-56127, Pisa, Italy; ‡ Instituto de Ciencia de Materiales de Madrid, Consejo Superior de Investigaciones Científicas, Sor Juana Inés de la Cruz 3, 28049 Madrid, Spain

**Keywords:** Josephson junctions, superconducting diode effect, Josephson diode, biharmonic drive, superconducting electronics

## Abstract

The superconducting diode effect has garnered significant interest due to its prospective applications in cryogenic electronics and computing, enabling directional supercurrent transport. This phenomenon has been demonstrated across various superconducting platforms, which typically necessitate unconventional materials with broken spatial symmetries or external magnetic fields, posing scalability and integration challenges. This work introduces an innovative method to realize the superconducting diode effect by disrupting spatiotemporal symmetries in a conventional Josephson junction utilizing a biharmonic alternating-current (AC) drive signal. We achieve wireless modulation of the diode’s polarity and efficiency with an antenna. Our findings indicate a diode efficiency reaching the ideal 100% over a broad frequency range, with a temperature resilience of up to 800 mK, and efficient AC signal rectification. This versatile and platform-independent superconducting diode signifies a promising component for advancing future superconducting digital electronics, including efficient logic gates, ultrafast switches, and dynamic half-wave supercurrent rectifiers.

## Graphical abstract

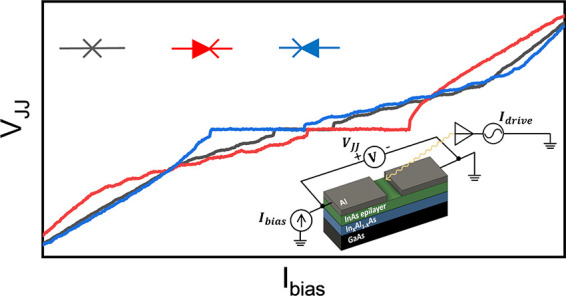

The superconducting diode effect has been extensively studied in recent years^1,2^ due to its potential applications in cryogenic electronics and dissipationless computation. Its zero resistance state, and thus zero energy loss, makes the superconducting diode a promising building block for future superconducting technologies,^3^ ultralow power consumption electronics, and high-frequency rectifiers.^4^ In fact, due to their nonreciprocal current–voltage characteristics, superconducting diodes can work as alternating-current (AC) signal rectifiers, the core of signal processing and power supply conversion. They have been either implemented in various platforms that break spatial symmetry via noncentrosymmetric materials^5–7^ or engineered in heterostructures.^8–10^ The superconducting diode effect was demonstrated in different spin–orbit interaction-based platforms under the action of a magnetic field,^5,9,11,12^ in field-free Josephson junctions (JJs),^6,13–15^ graphene-based systems,^16,17^ SQUIDs,^18,19^ and gate-tunable structures.^20–22^ Superconducting diodes were also made by integrating a superconductor with a magnetic atom on top of it,^23^ trapping the Abrikosov vortex,^24^ and Meissner screening.^25^ Lately, high-temperature superconducting diodes have been proposed, based on JJs made of twisted van der Waals bilayer^26^ or by cuprates.^27^ However, most of these devices require specially designed materials and heterostructures with broken spatial symmetries or the application of a magnetic field to break time reversal, making them difficult to scale and demanding integration into computer chips and quantum devices. Moreover, one of the significant limitations of superconducting diodes is their typically low rectification efficiency. To overcome this limitation, various strategies have been explored, including three-terminal JJ,^28^ radio-frequency-driven Josephson diodes,^19,29,30^ asymmetric interferometric devices,^31,32^ and periodically driven quantum-dot-based systems.^33,34^ Here, we demonstrate a superconducting diode based on broken spatiotemporal symmetries, induced by an external multiharmonic AC drive,^35^ applied to a conventional JJ. We experimentally implement such a scheme in an InAs-based JJ driven by a biharmonic drive: an AC drive signal composed of two frequency components, which can be galvanically injected into the JJ or irradiated by an antenna. The phase shift between the two harmonics controls the AC drive’s asymmetry and tunes the superconducting diode direction and efficiency. The antenna enables wireless control of the device, making the diode scalable, easy to reconfigure, and suitable for logic operations due to the fast tunability.^36^ Furthermore, because this scheme is independent of the specific design or structure of the device, our superconducting diode is compatible with various JJ platforms.

A sketch of the device, together with the diode idea and a first electrical characterization, is presented in Figure 1. As shown in Figure 1a, the device is placed in a four-terminal configuration under microwave irradiation (AC drive) provided by a broadband antenna placed a few millimeters away from the chip surface, as conventionally used in Shapiro experiments.^37–39^ Unlike the latter, we excite the antenna with a biharmonic signal composed of a superposition of two tones with different frequencies, amplitudes, and phases. The radiation emitted by the antenna is absorbed by the JJ, inducing an AC current of the form

1 $$I_{\mathrm{drive}}\left(t\right)=I_{1}\sin\left(2\pi f_{1}t\right)+I_{2}\sin\left(2\pi f_{2}t+\theta \right)$$

where *f*_2_ = 2*f*_1_ and *I*_1_ and *I*_2_ > 0 are the current amplitudes induced by the two tones. The phase shift θ can be adjusted so that the maximum (*I*_ac_^+^) and minimum (*I*_ac_^–^) of the AC drive are different (asymmetric drive): *I*_ac_^+^ ≠ |*I*_ac_^–^|, which is a sufficient condition to obtain nonreciprocity in the JJ^35^ with a maximum asymmetry obtained for θ = π/2 + *n*π (Figure 1b). The voltage/current (VI) characteristics of the JJ can be described by the phase dynamics with the resistively and capacitively shunted junction model:^40,41^

2 $$\frac{C\hbar }{2e}\mathrm{\phi ̈}+\frac{\hbar }{2eR_{N}}\mathrm{\phi ̇}+I\left(\phi \right)=I_{\mathrm{drive}}\left(t\right)+I_{\mathrm{bias}}$$

where ϕ is the phase, *C* and *R*_N_ are the capacitance and normal-state resistance of the JJ, *ℏ* is the reduced Planck constant, and *e* is the electronic charge. *I*(ϕ) = *I*_c_ sin­(ϕ) is the current-phase relationship (CPR), with *I*_c_ being the critical current of the JJ and *I*_bias_ the direct-current (DC) current bias. Within this model, the phase is a classical variable, equivalent to a particle under the action of a washboard potential that tilts in time according to the external drive: when |*I*_drive_| < *I*_c_, the phase particle is confined in a local minimum, whereas when |*I*_drive_| > *I*_c_, it is free to roll down the washboard potential, resulting in a nonzero voltage drop *V* = ℏϕ̇/2*e*. If the drive is asymmetric in direction, it is possible to achieve a nonzero voltage drop during the AC cycle only in one direction, if *I*_ac_^+^ < *I*_c_ < |*I*_ac_^–^|, as shown in Figure 1c. As a result, the VI curve of the JJ becomes asymmetric up to an ideal supercurrent diode effect, as illustrated in Figure 1d, where the supercurrent can flow only in one direction. Within the adiabatic approximation 2π*f*_1_ ≪ ω_c_, where ω_c_ = 2*eI*_c_ *R*_N_/*ℏ*, it is easy to show that the maximum supercurrent allowed in the forward/backward direction (*I*_c_^+^ and *I*_c_^–^, respectively) follows the simple relationship *I*_c_^±^ = ±(*I*_c_ – |*I*_ac_^±^|).^42^ When the diode effect occurs, the critical currents with positive or negative bias are different, namely, *I*_c_^+^ ≠ |*I*_c_^–^|. Obtaining an ideal diode requires max­(*I*_ac_^+^, |*I*_ac_^–^|) = *I*_c_. For the driving current in eq 1, this condition is achieved for |*I*_1_| + |*I*_2_| = *I*_c_ and θ = π/2 + *n*π. To quantify the diode effect, we use the diode efficiency η defined as $\eta =\frac{I_{c}^{+}-\left|I_{c}^{-}\right|}{I_{c}^{+}+\left|I_{c}^{-}\right|}$ , which equals 0 when there is no diode effect and ±1 in the ideal case (forward and backward diodes, respectively), corresponding to *I*_c_^–^ = 0 or *I*_c_^+^ = 0. Within the adiabatic approximation, we obtain $\eta =\frac{I_{\mathrm{ac}}^{+}-\left|I_{\mathrm{ac}}^{-}\right|}{I_{\mathrm{ac}}^{+}+\left|I_{\mathrm{ac}}^{-}\right|-2I_{c}}$ , which can be simplified into

3 $$\eta =\frac{\eta_{\mathrm{ac}}}{1-\frac{2I_{c}}{I_{\mathrm{ac}}^{+}+\left|I_{\mathrm{ac}}^{-}\right|}}$$

where $\eta_{\mathrm{ac}}=\frac{I_{\mathrm{ac}}^{+}-\left|I_{\mathrm{ac}}^{-}\right|}{I_{\mathrm{ac}}^{+}+\left|I_{\mathrm{ac}}^{-}\right|}$ quantifies the asymmetry of the drive. The equation shows that, even for nonfully asymmetric drives (η_ac_ < 1), it is possible to achieve ideal diodes (η = 1). Moreover, the latter expression is valid for any adiabatic drive beyond the biharmonic one and generic nonsinusoidal JJs. In the following, we implement this scheme for a semiconducting Al-InAs JJ fabricated on the InAs on an insulator (InAsOI) platform.^43–46^ The device is made of a 350-μm-thick GaAs (100) substrate, a 50 nm GaAs buffer, a 100 nm GaAs/AlGaAs superlattice, a 50 nm GaAs layer, a 1.25-μm-thick In_*X*_Al_1–*X*_As metamorphic buffer layer (*X* is from 0.15 to 0.81), a 100-nm-thick InAs intrinsically n-doped epilayer and two Al leads (100 nm of thickness and 5.6 μm of width) with interelectrode separation of 480 nm. Fabrication details can be found in previous works based on the same platform.^43–46^ To characterize the device and to ensure a good coupling between the microwave radiation and the JJ, a single drive with a frequency in the range of gigahertz is first applied to the antenna, showing clear Shapiro steps at different injected radio-frequency powers (*P*_RF_) and frequencies (*f*). Technical information about the experimental setup is reported in the Supporting Information, Methods section. Parts e and f of Figure 1 show the differential resistance d*V*/d*I* versus the DC bias current (*I*_bias_) and *P*_RF_ measured at 1.35 and 2.7 GHz frequencies, respectively. In the two cases, the characteristic Shapiro steps are visible in the zeros of the differential resistance represented by the darker regions of the plots. The corresponding plateaus are visible in Figure 1g, showing the VI characteristics measured at *P*_RF_ = −10 dBm for the two frequencies and compared to the gray curve obtained without microwave radiation. It is possible to estimate ω_c_ = 2π × 26 GHz from the latter, confirming that the drive is in the adiabatic regime. Precise integer Shapiro steps appear at voltage $nf\frac{h}{2e}$ , with *n* ranging from −10 to +10 (1.35 GHz) and from −6 to +6 (2.7 GHz); half-integer Shapiro steps^47^ are not observed, unveiling a negligible contribution of high-order harmonics in the CPR.^19,39^ Shapiro steps were visible up to 900 mK and at different frequencies ranging from 0.5 to 3 GHz, as reported in the Supporting Information, Methods and Additional Data sections. The damping of *I*_c_(*P*_RF_) is a consequence of the induced AC current and follows a square-root dependence in the power $I_{c}\left(P_{\mathrm{RF}}\right)=I_{c}\left(0\right)-I_{\mathrm{ac}}\left(P_{\mathrm{RF}}\right)=I_{c}\left(0\right)-Z\sqrt{P_{\mathrm{RF}}}$ , as highlighted by the green fits superimposed to the two colorplots. It is possible to extract the coupling parameter *Z*(*f*) from the fits, which enables conversion between the injected microwave power *P*_RF_ and the net AC current in the JJ.

**Figure 1:**
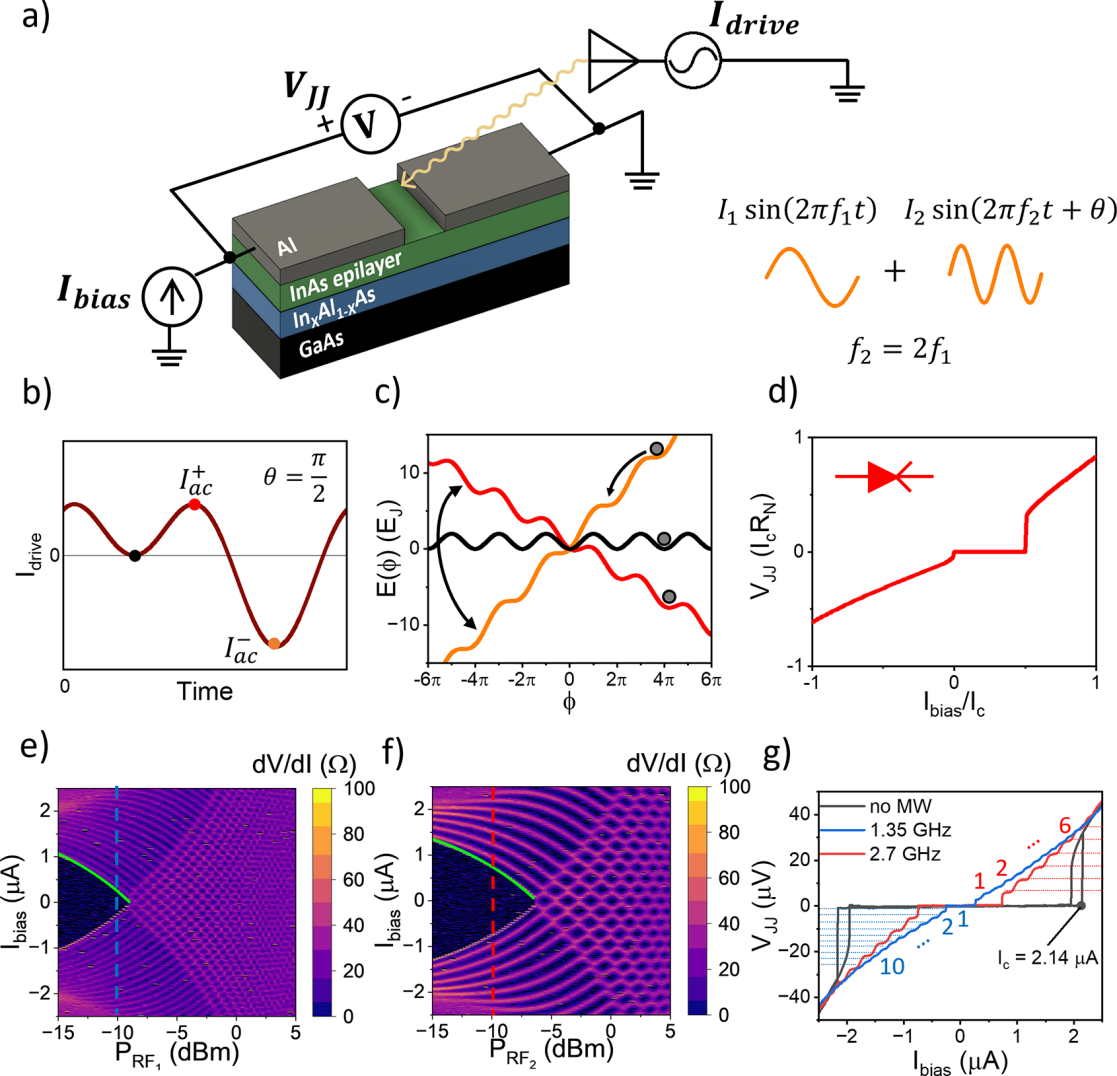
Concept device and precharacterization. (a) Schematic structure of the Al-InAs JJ and conceptual sketch of the setup. (b) Biharmonic drive signal applied to the antenna at a phase shift θ = π/2. (c) Dynamics of the washboard potential. Different colors refer to the selected time in part b. The tilt of the washboard potential evolves according to the instantaneous amplitude of the AC drive: zero (black curve), maximum *I*_ac_^+^ (red curve), and minimum *I*_ac_^–^ (orange curve). The gray dot represents the phase particle. (d) VI characteristics simulated from the drive in part b. At positive bias, below the critical current, the device is superconducting, while at negative bias, there is a nonzero voltage drop across the JJ. We set *R*_N_ = 10 *ℏ*/2*e* ^2^, *C* = 0.02*eI*_c_/*ℏ*, 2π*f*_1_ = 0.05ω_c_, and *I*_1_ = 2*I*_2_. (e–f) Differential resistance measured as a function of the current bias (*I*_bias_) and applied power (*P*_RF_) at *f* = 1.35 and 2.7 GHz showing the characteristic Shapiro plateaus. Green curves represent the fits of $I_{c}\left(P_{\mathrm{RF}}\right)=I_{c}\left(0\right)-Z\left(\omega \right)\sqrt{P_{\mathrm{RF}}}$ with *I*_c_(0) = 2.14 μA, $Z\left(1.35\;\mathrm{GHz}\right)=6\mu A/\sqrt{\mathrm{mW}}$ and $Z\left(2.7\;\mathrm{GHz}\right)=4.4\;\mu A/\sqrt{\mathrm{mW}}$ . (g) VI curves with drive at *P*_RF_ = −10 dBm and *f* = 1.35 and 2.7 GHz (blue and red, respectively, corresponding to the dashed lines in part e–f) and no microwave applied (gray). The numbers denote the Shapiro steps observed at $\Delta V=nf\frac{h}{2e}$ . From the gray curve, we estimate *I*_c_ = 2.14 μA, *R*_N_ ≈ 26 Ω, and *I*_c_ *R*_N_ ≈ 55 μV. Measurements are taken at *T* ≈ 70 mK.

The reciprocal VIs shown in Figure 1g evolve into a nonreciprocal one under the simultaneous action of the two microwave signals. This is demonstrated in Figure 2a, which illustrates the VI curves measured under the action of the biharmonic microwave signal (eq 1) with θ = ±π/2. As predicted by the model, θ determines the direction and efficiency of the diode: it can be controlled from ideal (η = ±1 in θ = ±π/2) to no (η = 0 in θ = 0) diode. The ideal diode was obtained by adjusting θ and the amplitude of the microwave drive, in good agreement with what was predicted in ref 35. Moreover, signatures of the two combined Shapiro steps are still visible at finite voltages. It is worth noting that, due to the asymmetry of the drive, Shapiro steps are mainly visible on the resistive side of the diode, where the microwave signal is stronger. This asymmetric behavior is further illustrated in Figure 4 of the Supporting Information, showing Shapiro steps at different combinations of *P*_RF_1__ and *P*_RF_2__. The driving signal was also monitored from the source at different θ, confirming the biharmonic shape of the drive, as shown in Figure 2b. In Figure 2c, we show the evolution of η at different *P*_RF_1__, while keeping *P*_RF_2__ fixed and θ = π/2. Notably, the ideal diode behavior occurs at *P*_RF_1__ = −7 dBm, corresponding to the condition |*I*_ac_^–^(*P*_RF_1__,*P*_RF_2__)| = *I*_c_. η decreases monotonically by lowering *P*_RF_1__, while maintaining the condition |*I*_ac_^–^(*P*_RF_1__,*P*_RF_2__)| < *I*_c_. For values above −7 dBm, the drive amplitude becomes so large that the supercurrent was suppressed. The diode effect was tested at different combinations of *P*_RF_1__ and *P*_RF_2__, and the behavior of the diode efficiency as a function of the driving amplitudes was computed, as reported in Shapiro steps with a biharmonic drive and diode efficiency at different *I*_1,2_ subsections of the Supporting Information. Observing the diode effect with a biharmonic drive is also possible on a different platform, such as a SNS JJ, as reported in the Supporting Information, where nonreciprocal VI curves for a Nb/Au/Nb JJ are presented. Additionally, the biharmonic-driven diode effect is not limited to the gigahertz frequency range, where Shapiro steps can be observed, but is visible within a broader range of frequencies. Indeed, we tested it at different drive frequencies within the adiabatic approximation, ranging from 100 Hz to a few gigahertz. In Figure 3a, a megahertz drive is applied through the antenna: we observe a tunable diode effect at *f*_1_ = 700 MHz with negligible Shapiro steps, as shown in Figure 3b. To prove the validity of the effect also at lower frequencies (*f*_1_ ≪MHz), the driving signal is galvanically injected into the junction according to the scheme presented in Figure 3c because the physical dimensions of the antenna limit the frequency bandwidth to 0.5–5 GHz (see the Supporting Information, Methods section). Adjusting the phase shift and the amplitude of the signal, we also achieve η = 1 for *f*_1_ = 1 kHz and *f*_1_ = 100 Hz, as shown in parts d and e of Figure 3, respectively. In Figure 3f, η is quantified as a function of θ for the 100 Hz biharmonic driving signal, demonstrating the continuous tunability of the effect. Moreover, the effect of the diode is not limited by the choice of the second harmonic (*f*_2_ = 2*f*_1_). Still, it can be easily demonstrated that asymmetric drives are possible for all harmonics (*f*_2*n*_ = 2*nf*_1_). In Figure 3g, we evaluate η obtained at different *f*_2*n*_ for fixed *I*_1_ and *I*_2_ optimized for *f*_2_ = 2*f*_1_. The system is reciprocal for odd harmonics because η_ac_(2*n* + 1) = 0, while η is reduced for higher even harmonics. The reduction is due to a decrease in the asymmetry of the drive for higher-order harmonics, η_ac_(2*n*), which requires an enhanced driving signal to reach η = 1 for the chosen parameters. Detailed information about the harmonic dependence of the driving signal asymmetry is provided in the Supporting Information, Additional Data section.

**Figure 2:**
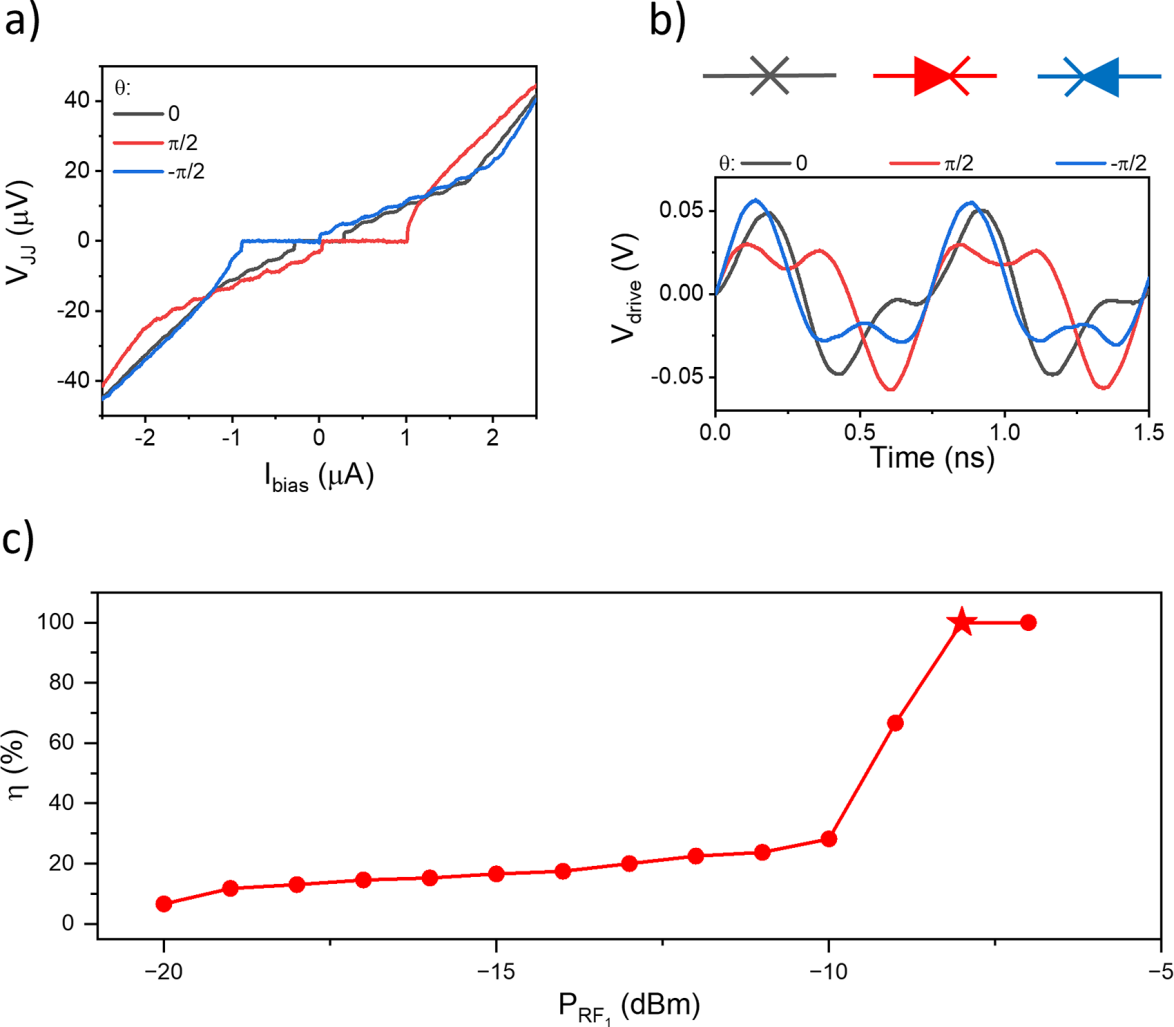
Biharmonic drive diode. (a) VI curve under applied biharmonic drive (eq 1) with *f*_1_ = 1.35 GHz and *f*_2_ = 2.7 GHz at different θ, *P*_RF_1__ = −8 dBm and *P*_RF_2__ = −10 dBm. (b) Diode symbols and drive voltage monitored from the generator output: no diode (gray curve), positive diode (red curve), and negative diode (blue curve). (c) Diode efficiency at different *P*_RF_1__, *P*_RF_2__ = −10 dBm, and θ = π/2. The star corresponds to the η estimated from the red curve in part a. Measurements are taken at *T* ≈ 70 mK.

**Figure 3:**
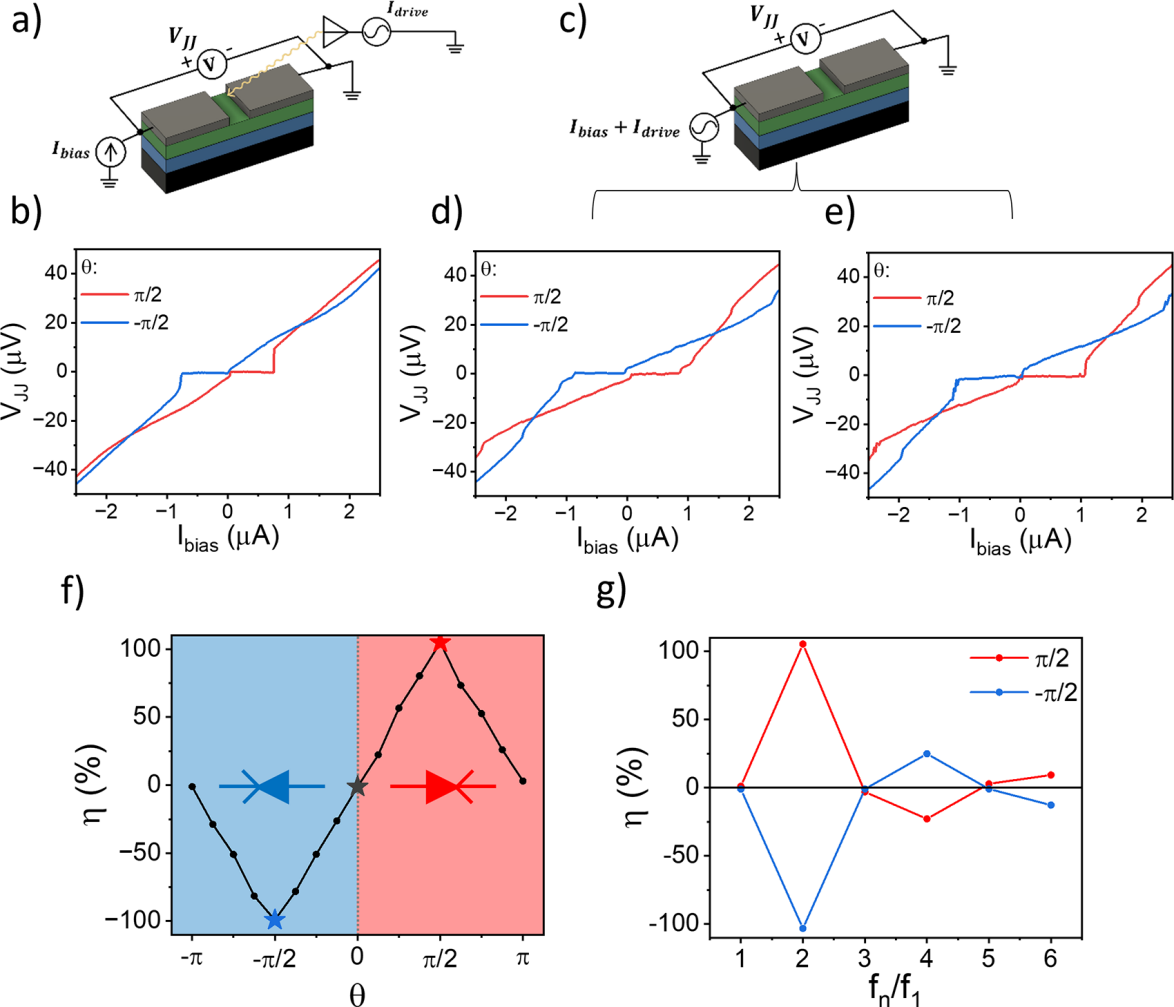
Diode with biharmonic drive at different frequencies. (a) Sketch of the experimental setup with the driving signal injected through the antenna, with *P*_RF_1__ = *P*_RF_2__ = 5.75 dBm. (b) VI curve under an applied biharmonic drive with *f*_1_ = 700 MHz at θ = ±π/2. (c) Sketch of the experimental setup with the driving signal injected through the JJ. *I*_1_ = *I*_2_ and different peak-to-peak amplitude *I* ^pp^ ≡ *I*_ac_^+^ – *I*_ac_^–^. (d and e) VI curves under applied biharmonic drive with θ = ±π/2, *f*_1_ = 1 kHz (*I* ^pp^ = 4.4 μA), and *f*_1_ = 100 Hz (*I* ^pp^ = 3.8 μA), respectively. (f) Diode efficiency vs θ for a low-frequency biharmonic drive used in part f. The blue and red parts refer to the direction of the diode. Colored stars indicate the ideal cases in part f. (g) Diode efficiency at different *f*_*n*_/*f*_1_ (*f*_1_ = 100 Hz drive and *I* ^pp^ = 3.8 μA) for θ = π/2 (red curve) and −π/2 (blue curve). Measurements are taken at *T* ≈ 70 mK.

A testbed application of the diode as a rectifier is shown in Figure 4. Here, a low AC bias current (*f*_AC_ = 10 Hz ≪ *f*_1_) is injected into the JJ (Figure 4b), and the output voltage is measured in real time, as shown in Figure 4c,d. According to the direction of the diode, tuned by θ, we observe positive (negative) half-wave rectification of the output voltage, as shown in Figure 4c, for the red (blue) curve. Electrical rectification is not observed at θ = 0 despite deformation of the AC output signal due to the nonlinear response of the JJ with zero output voltage when the current bias is between *I*_c_^–^ and *I*_c_^+^ (Figure 4d). Moreover, continuous control over θ allows intermediate rectification schemes above half-wave rectification. A full-wave rectifier can be realized by combining multiple superconducting diodes in a bridge structure.^48,49^ Other tested bias frequencies ranging from 30 to 300 Hz are reported in the Supporting Information, Additional Data section. The current rectification range of this scheme is limited by *I*_c_, and in the ideal case (η = 1), this is additionally reduced to |*I*_s_*| < *I*_c_ defined as the critical current in ideal rectification |*I*_s_*|= *I*_c_ - min­(*I*_ac_^+^,|*I*_ac_^–^|), as shown in Figure 5a. Because an ideal diode efficiency is achieved when the peak amplitude of the drive matches the critical current, i.e., max­(|*I*_ac_^±^|) = *I*_c_, variations in *I*_c_, which depend on the temperature, external magnetic field, and junction length,^42^ will affect the diode efficiency. If the drive is fixed, then the efficiency will naturally evolve in response to changes in *I*_c_. A discussion on the temperature dependence of η ≤ 1 is included in the Supporting Information, Additional Data section.

**Figure 4:**
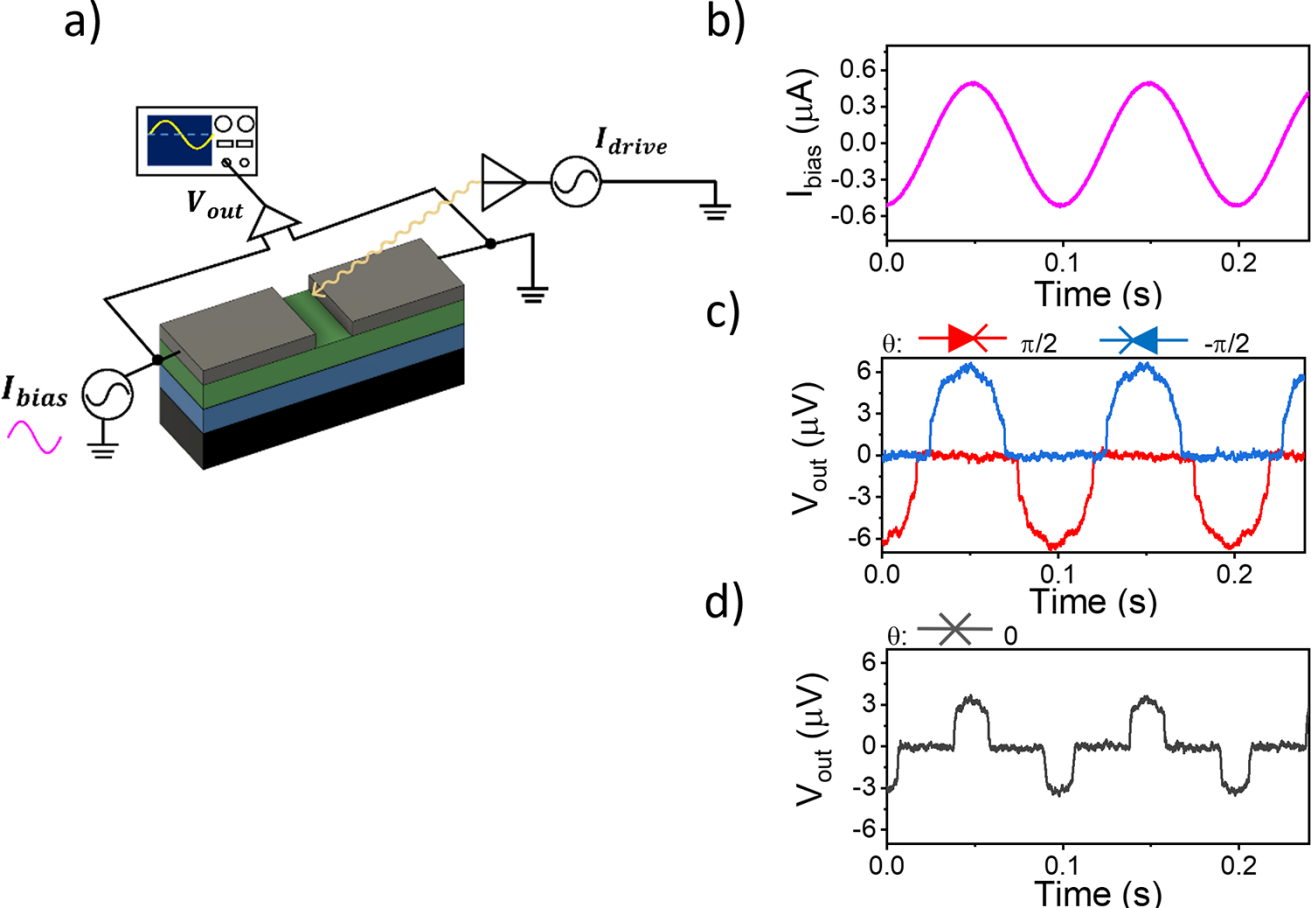
Rectification of an AC signal. (a) Sketch of the experimental setup with the driving signal (*f*_1_ = 1.35 GHz, *f*_2_ = 2*f*_1_, and emitted power *P*_RF_1__ = −8 dBm and *P*_RF_2__ = −10 dBm) through the antenna. The output voltage is real-time measured with an oscilloscope and averaged over 128 waveforms. (b) Sinusoidal bias signal of *f*_AC_ = 10 Hz and *I*_bias_^pp^ = 1 μA injected through the JJ. (c) Half-wave rectified signals. (d) Output signal when no diode effect occurs. Measurements are taken at *T* ≈ 70 mK.

**Figure 5:**
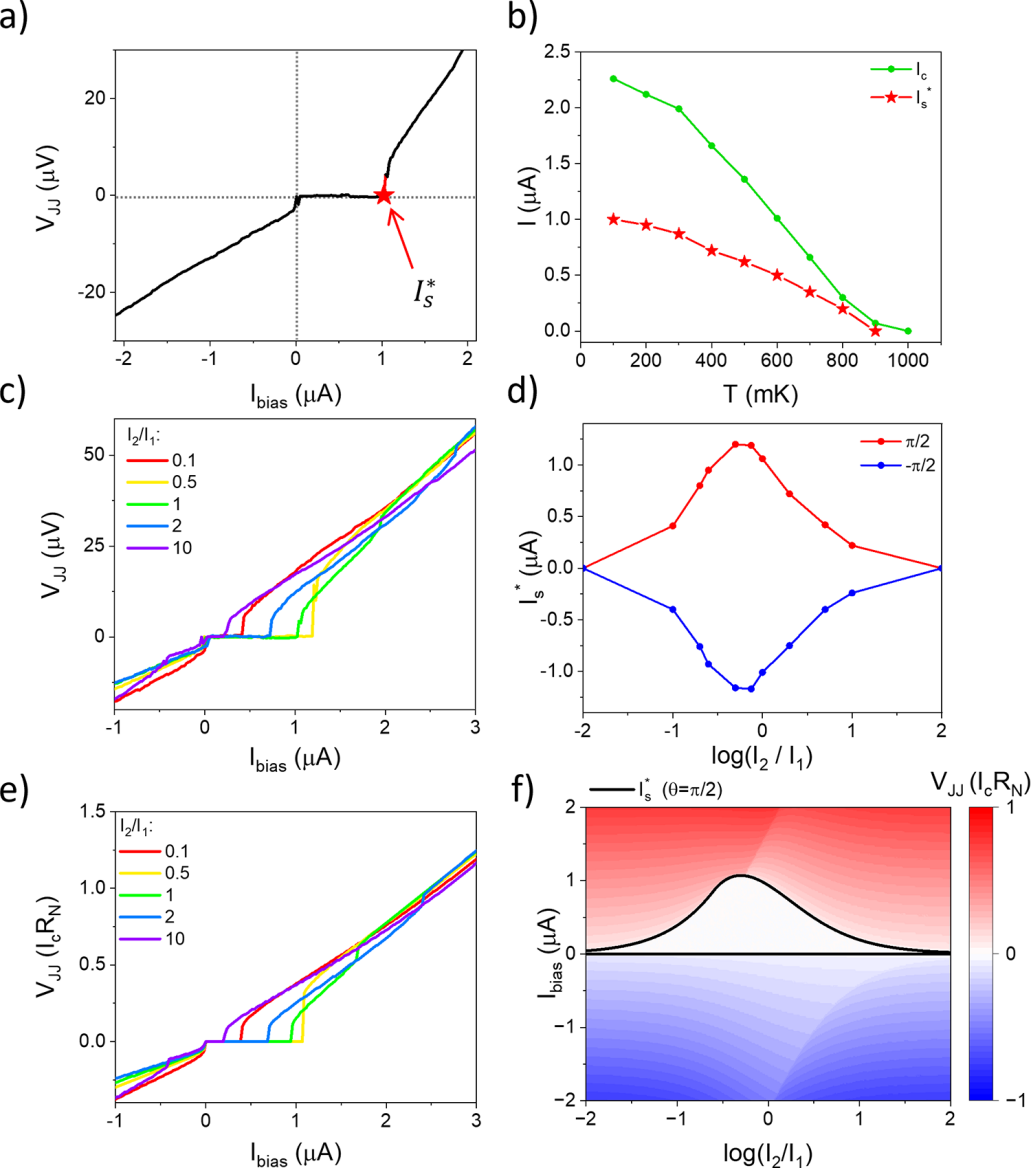
Range of operation and limitations: role of temperature and*I*_2_/*I*_1_. (a) VI curve for an ideal diode (driving frequency *f*_1_ = 100 Hz, *I* ^pp^ = 3.8 μA, and *I*_1_ = *I*_2_): *I*_s_* is highlighted in red. (b) Critical current *I*_c_ (green curve) and *I*_s_* (red curve) as a function of the temperature. For the *I*_s_* temperature dependence, a 100 Hz biharmonic drive signal with *I*_1_ = *I*_2_ at different amplitudes is used. *I*_s_* is zero for 900 mK. (c and d) VI curve (c) and *I*_s_* (d) as a function of *I*_2_/*I*_1_ for *f*_1_ = 100 Hz. To achieve an ideal diode effect, *I* ^pp^ = 4.4, 3.6, 3.8, 4.2, and 4.6 μA respectively for *I*_2_/*I*_1_ = 0.1, 0.5, 1, 2, and 10. (e and f) Simulated VI curve (e) and colormap (f) as a function of *I*_2_/*I*_1_. For the simulations, we set *R*_N_ = 10*ℏ*/2*e* ^2^, *C* = 0.02*eI*_c_/*ℏ*, and 2π*f*_1_ = 0.05ω_c_. The black curve in part f is the analytical model, where we set *I*_ac_^–^ = *I*_c_, and the phase boundary is given by *I*_c_ – *I*_ac_^–^. Measurements for parts c and d are taken at *T* ≈ 100 mK.

The temperature dependence of *I*_s_* naturally follows the damping of *I*_c_, the critical current of the JJ without any applied drive, as reported in Figure 5b. Based on these results, our diode can operate up to temperatures of ∼800 mK, where signals of up to *I* ^pp^ = 0.5 μA peak-to-peak can be rectified. We refer to the ability to achieve 100% diode efficiency even in proximity to the JJ transition temperature as a temperature-resistant behavior. Larger critical currents can be achieved in systems with larger *I*_c_, and the limiting temperature depends on the critical temperature of the superconductor and the device geometry. Therefore, the choice of other superconductors can increase the diode operating conditions. The impact of the *I*_2_/*I*_1_ ratio on *I*_s_* is investigated in Figure 5c, showing the VI characteristics measured at different *I*_2_/*I*_1_. The maximum *I*_s_* is achieved for *I*_2_/*I*_1_ = 0.5, as predicted by perturbation theory,^35^ and suggests a symmetry point around this value, as outlined in Figure 5d, where *I*_s_*­(*I*_2_/*I*_1_) is extracted for θ = ±π/2. The diode effect monotonically decreases for larger and smaller ratios. Figure 5e shows the simulated VI curves at different *I*_2_/*I*_1_ ratios, confirming the maximum at *I*_2_/*I*_1_ = 0.5 and the behavior of *I*_s_*­(*I*_2_/*I*_1_), as reported in Figure 5f (black curve).

It has been demonstrated that a biharmonic drive induces a tunable diode effect within a hybrid JJ, which requires fewer complexities or asymmetries in the system. Using an antenna enables wireless, fast, and simultaneous control over the diode’s direction and efficiency by modifying the driving tones’ relative phase. Moreover, the diode exhibits additional tunability by adjustment of the relative amplitudes of the two tones. This diode showcases exceptional adaptability, achieving 100% efficiency over the drive’s broad frequency spectrum, from a few hertz to gigahertz, and different combinations of even harmonics. The tunable Josephson diode has effectively rectified AC signals across frequencies and temperatures of up to 800 mK, limited only by the superconductor’s critical temperature. The rectification range was tested and optimized for biharmonic drive with a different ratio between the first- and second-harmonic amplitudes.

In summary, we have developed a highly versatile, temperature-resistant, and platform-agnostic superconducting diode that is straightforward to manufacture and can be tuned with easily generated and controlled signals. Notably, the dynamically adjustable polarity of the superconducting diode could facilitate the development of fundamental logic gates and ultrafast switches, thereby advancing superconductor-based digital electronics. Additionally, the capability of this superconducting diode to function as a controllable active rectifier, essential for the conversion and management of electrical energy, paves the way for advances in superconducting integrated power electronics. Such supercurrent-based rectifiers fundamentally differ from traditional semiconductor rectifiers, leading to a reduced power dissipation. Future investigations will explore the diode in the fast nonadiabatic regime 2π*f* ∼ ω_c_, an unresolved issue that could extend its operating speed.

## Supplementary Material



## Notes

Further information on device fabrication, experimental setup, and additional measurements studying power, temperature, and platform dependencies (PDF)
