# Supplementary material for: Biharmonic-Drive Tunable Josephson Diode
Source: Nano Lett. 2025 Sep 17;25(39):14451–8. doi: 10.1021/acs.nanolett.5c03922 (PMC12492391; doi:10.1021/acs.nanolett.5c03922)
Supplement: Supplementary file 1 [file nl5c03922_si_001.pdf]

Supporting Information for Biharmonic-drive tunable Josephson diode

Laura Borgongino,[*,†] Rubén Seoane Souto,[‡] Alessandro Paghi,[†] Giulio Senesi,[†] Katarzyna Skibinska,[†] Lucia Sorba,[†] Elisa Riccardi,[†] Francesco Giazotto,[†] and Elia Strambini[*,†]

†*NEST, Istituto Nanoscienze-CNR and Scuola Normale Superiore, I-56127, Pisa, Italy*

‡*Instituto de Ciencia de Materiales de Madrid (ICMM), Consejo Superior de Investigaciones Científicas (CSIC), Sor Juana Inés de la Cruz 3, 28049 Madrid, Spain*

E-mail: laura.borgongino@sns.it; elia.strambini@cnr.it

Methods

Sample information

The InAsOI heterostructure was grown on GaAs (100) substrate with a Molecular Beam Epitaxy technique. Starting from the 350 μm-thick semi-insulating GaAs (100) substrate, the sample consists of: a 50 nm GaAs buffer, a 100 nm GaAs/AlGaAs superlattice, a 50 nm GaAs layer, a 1.25 μm-thick step-graded $In_X Al_{1-X} As$ metamorphic buffer layer (X from 0.15 to 0.81) that acts as insulator at cryogenic temperature and a 100 nm-thick InAs semiconductive epilayer. This sample structure was adapted from Refs.[1,2] Classical Hall effect measurements in Hall bar configuration have been used to estimate the sheet carrier concentration and mobility of this sample, respectively: $n_{2D} = 1.94 \times 10^{12}\text{cm}^{-2}$ and $\mu_n = 8.5 \times 10^3$ cm^2/Vs at 3K. InAsOI substrates were cleaned (ACE and IPA), passivated with a $(NH_4)_2S_x$ solution and loaded in an electron beam evaporator to evaporate 100 nm of Al all over the sample. In order to define the MESA, an UV-lithography was performed: then, after developing in MF319, the exposed Al layer was etched in Al Etchant Type D and the exposed InAs epilayer was etched in a $H_3PO_4 : H_2O_2$ solution. To define the Josephson junction length, it was necessary to define EBL-markers and deposit a 10/50 nm-thick Ti/Au bilayer in a thermal evaporator; then the Al contacts on the InAs MESA were defined by marker-aligned EBL and selected chemical etching of the Al. Additional information on device fabrication can be found in.[1,3–5]

Low temperature DC and AC setup

The device was mounted on a chip in a Leiden cryostat (CF-CS110) equipped with a magnet, cooled down to the base temperature (70 mK). A four-wire configuration was used to carry out the electrical characterization (VI curves) of the device: the junction was current-biased using a Yokogawa GS200 voltage source over a 1 MΩ resistor, the voltage V_{JJ} across the junction was amplified (Voltage Amplifier DL1201) and measured with an Agilent 34401A

with NPLC=1. An out-of-plane magnetic field of 60 μT was used only to maximize the switching current and maintained throughout all the measurements: we used a Yokogawa GS200 voltage source over a 100 Ω resistor connected to an American Magnetics magnet inside the cryostat. Microwave drives generated from two Anritsu signal generators (68369B and MG3694A) were summed using a DC pass power splitter/combiner (2-10 GHz, 50 Ω) and applied to an open-ended coaxial cable near the device. By fixing the power and varying the frequency, we show that only some emitted frequencies "match" with the response of the device, showing a change in the VI characteristic, as reported in Figure 1a: here, the stripes represent a damping of the critical current or the appearance of Shapiro steps. This allowed the selection of the frequencies that optimized the coupling between the antenna and the sample. Additionally, to characterize the reflection behavior of the antenna we measured with a Vector Network Analyzer the return loss S_{11}, as shown in Figure 1b. The attenuation of the microwave signal due to cables is estimated through the S_{21} parameter analysis in frequency, as reported in Figure 1c: it shows that there is an attenuation of -100 dBm above 5 GHz, so our operational range is below 5 GHz. For the measurements in Hz and kHz regime, the driving signals were applied with an arbitrary waveform generator (Agilent 33220A) over a 1 MΩ resistor, the flowing current was amplified and collected with an Agilent 34401A with NPLC=1. The real-time measurements were carried out with a similar four-wire technique, using an arbitrary waveform generator (Agilent 33220A) as a source; the voltage drop over the junction and the flowing current were amplified and acquired using a Tektronix TDS 2024B oscilloscope (128 means). The cabling inside the dilution refrigerator is shown in Figure 2: there is a low-pass frequency stage between the Mixing Chamber and the sample.

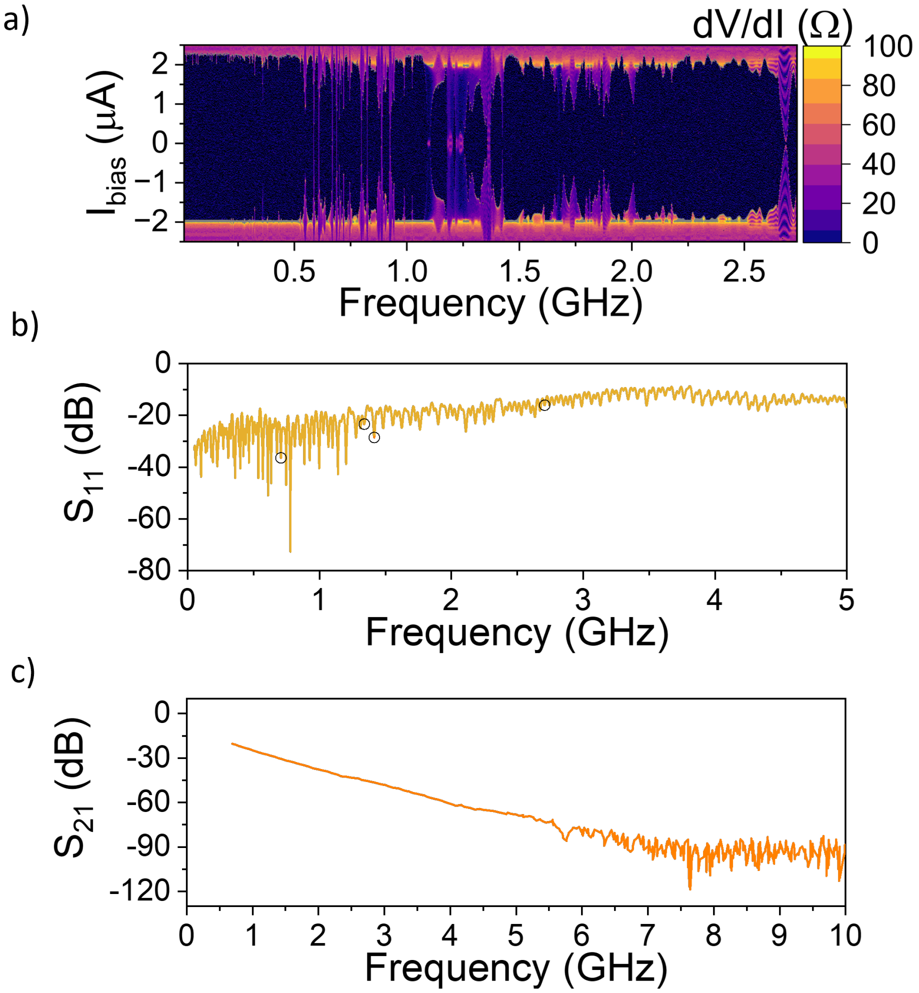

Figure 1: **Frequency matching of the antenna.** a) Evolution of differential resistance as a function of current bias I_{bias} and frequency at applied power $P_{RF} = -10$ dBm. b) Reflection coefficient S_{11} as a function of the antenna frequency. Circles indicate the chosen frequencies related to the biharmonic-drive diode plots. c) Power received by the antenna relative to the power emitted S_{21} as a function of the antenna frequency.

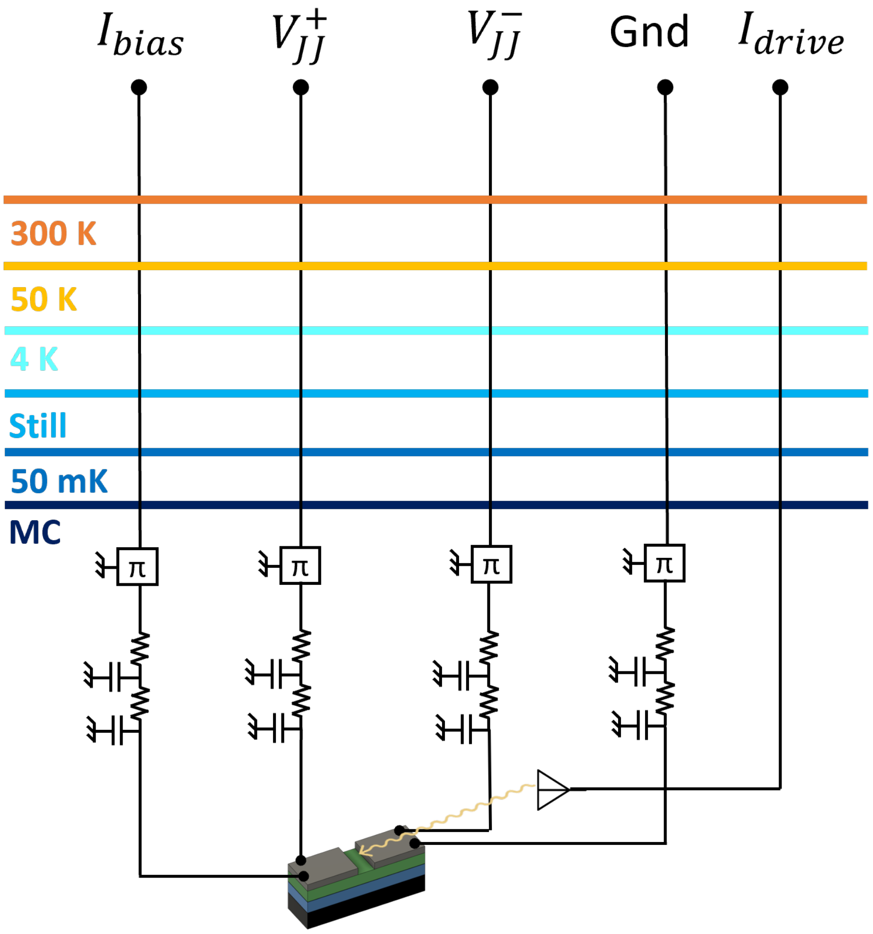

Figure 2: **DC and microwave setup.** Schematic of the cabling configuration within the dilution refrigerator. RC filters have resistance 1.1 kΩ and capacitance 10 nF: they constitute a low-pass filter block with a cutoff of 10 kHz.

Additional data

Shapiro steps in temperature

Shapiro steps were obtained by sweeping the current bias I_{bias} from negative to positive. Here we display the measurements for a single frequency drive of 2.7 GHz at different temperature (Figure 3): Shapiro steps are still observed at 800 mK (Figure 3g).

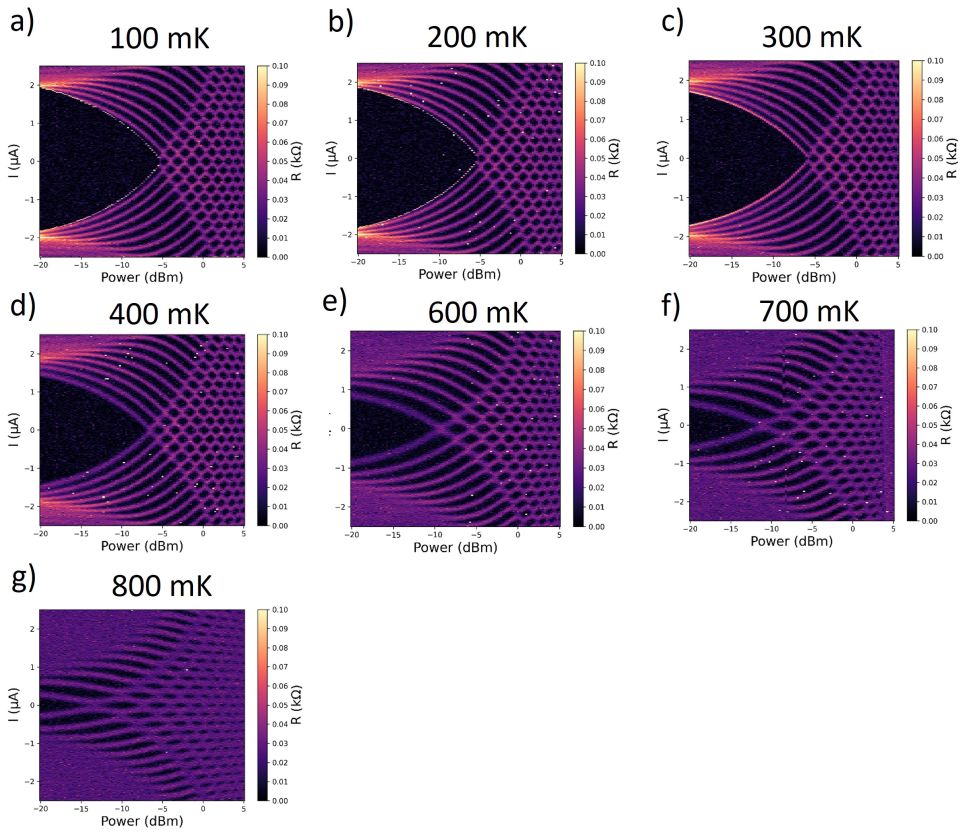

Figure 3: **Shapiro steps in temperature (f = 2.7 GHz).** a-g) Shapiro evolution of differential resistance as a function of current bias (I_{bias}) and applied power (P_{RF}) at $f = 2.7$ GHz at different temperatures.

Shapiro steps with a biharmonic drive

Shapiro steps are also visible when a biharmonic drive (with $\theta = -\pi/2$) is applied. In Figure 4 we show the evolution of the differential resistance as a function of the applied power P_{RF_2}

at different P_{RF_1}.

Diode efficiency at different $I_{1,2}$

Figure 5 illustrates η versus the ratio $(I_1/I_2)^2$ with I_2 held constant and $\theta = \pi/2$. The qualitative trend of η with respect to $(I_1/I_2)^2$ mirrors the behavior expected as a function of power, assuming a monotonic but nonlinear relationship between power and current and captures the key features of the diode efficiency at different driving amplitudes.

Biharmonic drive diode on a different platform (Nb/Au/Nb JJ)

The biharmonic-drive diode can be realized on diverse material platforms and does not rely on material-specific spin-orbit interactions or particular device geometry. To further prove the platform-independency of our approach, we performed additional measurements on a Nb/Au/Nb SNS JJ. Figure 6a shows the VI curves measured under a biharmonic drive with $\theta = \pm\pi/2, 0$, demonstrating the diode effect. The direction and efficiency of the diode are controlled by θ ranging from ideal ($\eta = \pm 1$ in $\theta = \pm\pi/2$) to zero ($\eta = 0$ in $\theta = 0$). Figure 6b shows the evolution of η with P_{RF_1}, keeping P_{RF_2} fixed and $\theta = \pi/2$.

Harmonic dependence of signal asymmetry

When considering higher order harmonics in the driving signal, the diode efficiency η decreases due to the reduction of the drive asymmetry quantified by η_{ac}, which is directly proportional to η. As the harmonic order n increases, the waveform extrema become more symmetric, leading to a suppression of η_{ac} and a sign reversal. Figure 7 illustrates this behavior for $2n = 2, 4, 6$, showing the corresponding drive waveforms and calculated η_{ac}. Increasing the drive amplitudes $I_{1,2}$ can restore the asymmetry for higher harmonics up to the ideal case.

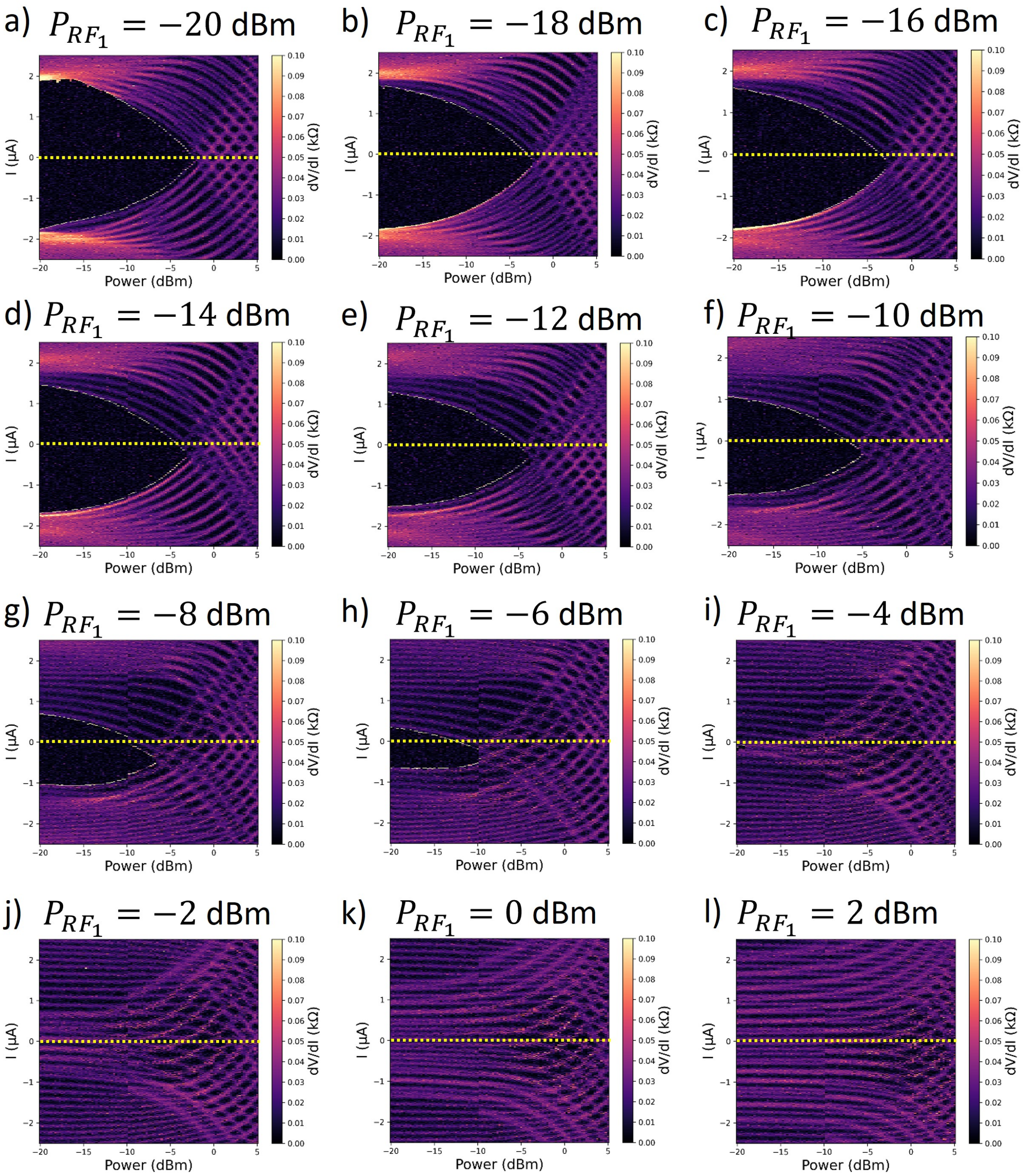

Figure 4: **Shapiro steps with biharmonic drive.** a-l) Shapiro evolution of differential resistance as a function of current bias (I_{bias}) and applied power P_{RF_2} at $f_1 = 1.35$ and $f_2 = 2.7$ GHz at different I_1 amplitudes.

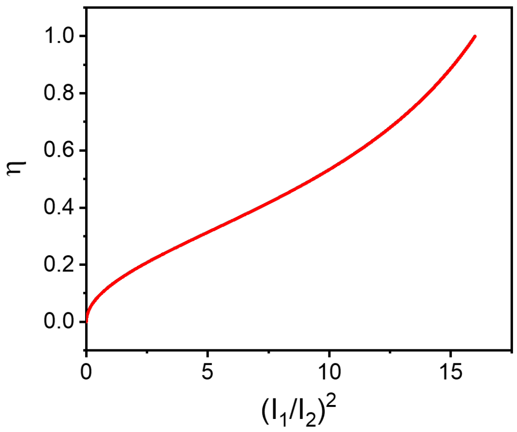

Figure 5: **Diode efficiency as a function of biharmonic signal amplitudes**. η at different $(I_1/I_2)^2$ ratio, for a biharmonic signal with $\theta = \pi/2$, $f_1 = 1.35$ GHz and $f_2 = 2.7$ GHz. I_2 is fixed at 0.2 μA.

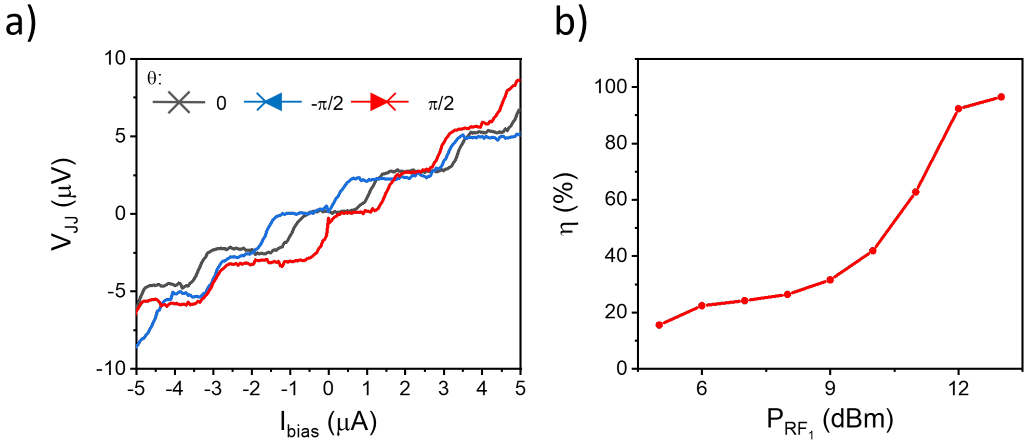

Figure 6: **Biharmonic drive diode for Nb/Au/Nb JJ.** a) VI curve under applied biharmonic drive with $f_1 = 1.35$ GHz and $f_2 = 2.7$ GHz at different θ, $P_{RF_1} = 13$ dBm and $P_{RF_2} = 0$ dBm. b) Diode efficiency at different P_{RF_1}, $P_{RF_2} = 0$ dBm and $\theta = \pi/2$. Measurements are taken at $T \approx 950$ mK.

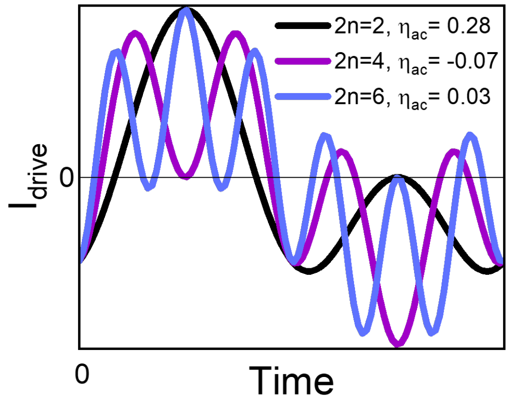

Figure 7: **Harmonic dependence of signal asymmetry** Biharmonic drive signal $I_{drive}(t) = I_1 \sin(2\pi f_1 t) + I_2 \sin(2\pi(2nf_1)t - \pi/2)$ and calculated asymmetries η_{ac} for $2n = 2, 4, 6$, $I_1 = I_2$, $I^{pp} = 3.8$ μA.

Rectification of AC signals

The biharmonic-drive diode was used to rectify AC signals: Figure 8 shows the biharmonic-drive diode as rectifier for bias signals ranging from 30 to 300 Hz. At higher frequency (Figure 8e) the output signal is distorted due to the filtering stage in the electrical setup.

Role of I_2/I_1

Figure 9 depicts the dependence of the diode efficiency η with respect the ratio I_2/I_1, that represent the weight of the second harmonic over the first harmonic in the driving signal. The efficiency η is analyzed as a function of peak-to-peak current (I^{pp}) that we experimentally controlled, once fixed the I_2/I_1 ratio.

Temperature dependence of the diode efficiency

According to

$$\eta = \frac{\eta_{ac}}{1 - \frac{2I_c}{I_{ac}^{+} + |I_{ac}^{-}|}}, \tag{1}$$

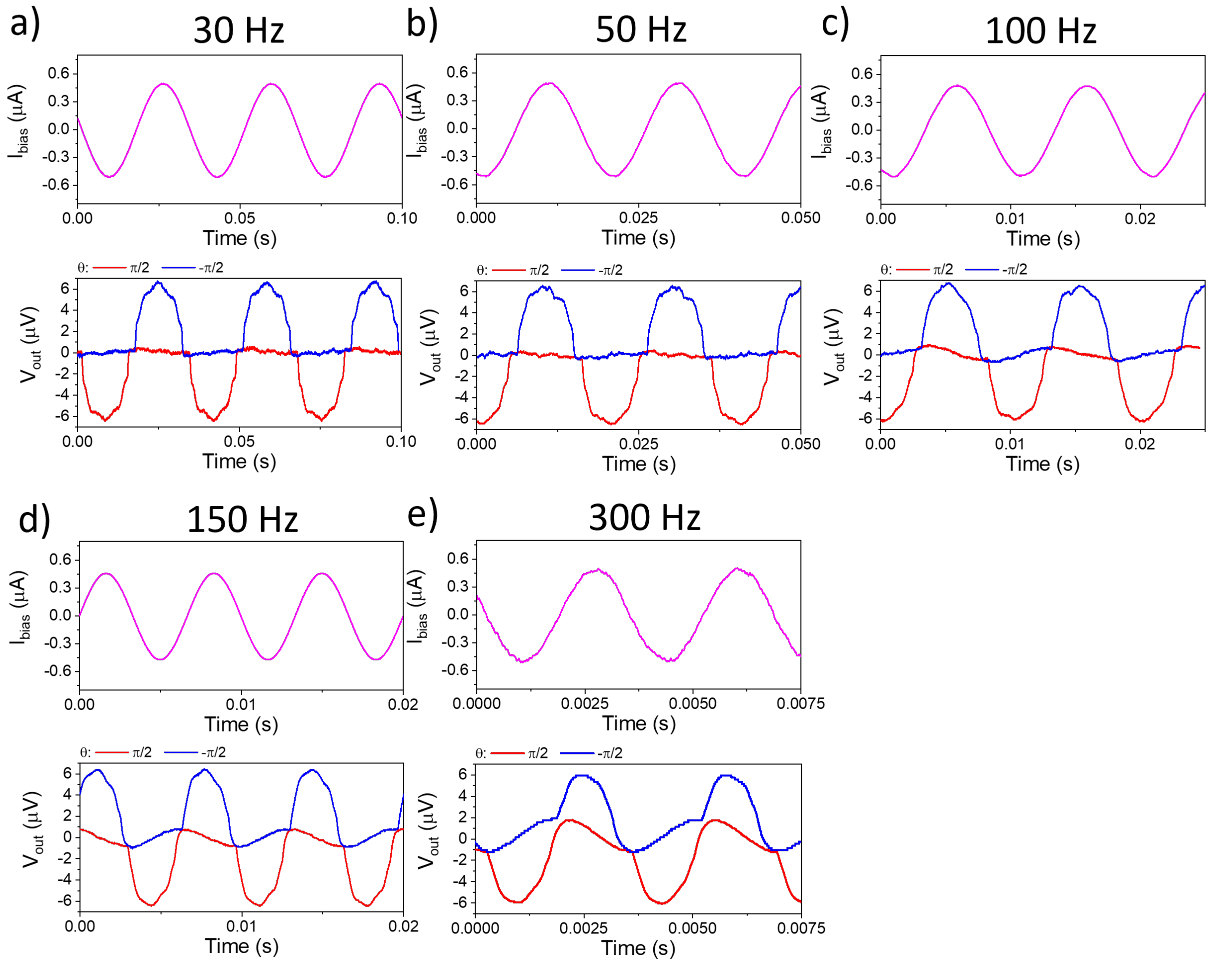

Figure 8: **Rectification of AC signal (more bias frequencies).** a-e) Sinusoidal bias signal of $f_{AC} = 30 - 300$ Hz and $I_{bias}^{pp} = 1$ μA injected through the JJ and half-wave rectified signal (blue and red curves). The drive signal has$f_1 = 1.35$ GHz, $f_2 = 2f_1$ and emitted power $P_{RF_1} = -8$ dBm and $P_{RF_2} = -10$ dBm

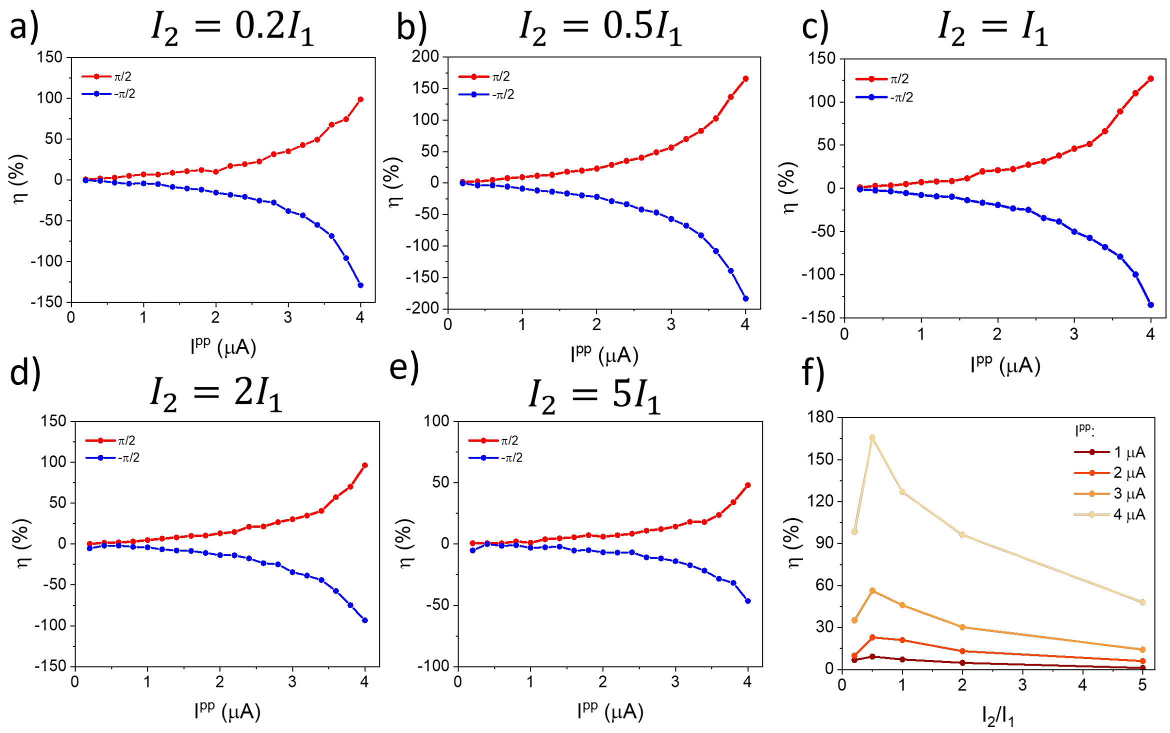

Figure 9: **Diode efficiency at different I_2/I_1.** a-e) η vs I^{pp} of the driving signal. f) η vs I_2/I_1 at various I^{pp} of the driving signal. Drive frequency $f_1 = 100$ Hz.

the diode efficiency η naturally varies with temperature, because $I_c(T)$. Figure 10a and Figure 10b respectively illustrate $\eta(T)$ and $\eta(I_c)$, using a biharmonic drive calibrated to yield a diode efficiency of 100% at 100 mK and the experimental data of $I_c(T)$. For a fixed temperature (and therefore for a fixed I_c), the diode efficiency can be tuned by changing the driving parameters as illustrated in Figure 10, which shows $\eta(I_c)$ for different $I^{pp} = I^+_{ac} + |I^-_{ac}|$ of the driving signal. For a fluctuation $\Delta T = 100$ mK around 400 mK the critical current of the device changes approximately of $\Delta I_c \approx 0.6$ μA. As shown in Figure 10d, different choices of the driving parameters will result in different values of $\Delta\eta$, showing that an appropriate selection of the driving parameters can effectively mitigate the sensitivity of η to fluctuations in temperature and I_c enhancing operational stability and robustness of the diode. Additionally we conducted another experiment with a different device consisting in a Nb/Au/Nb JJ, for which we investigated intermediate configurations of the diode effect. In Figure 11a and Figure 11b, we show experimental and computed

data for $\eta(T)$ and $\eta(I_c)$, respectively. This time, we consider an intermediate diode configuration, so that at 200 mK the diode efficiency is 35%. Also for this device we show how η evolves with the driving parameters (Figure 11c) and with fluctuations of I_c (Figure 11d)."

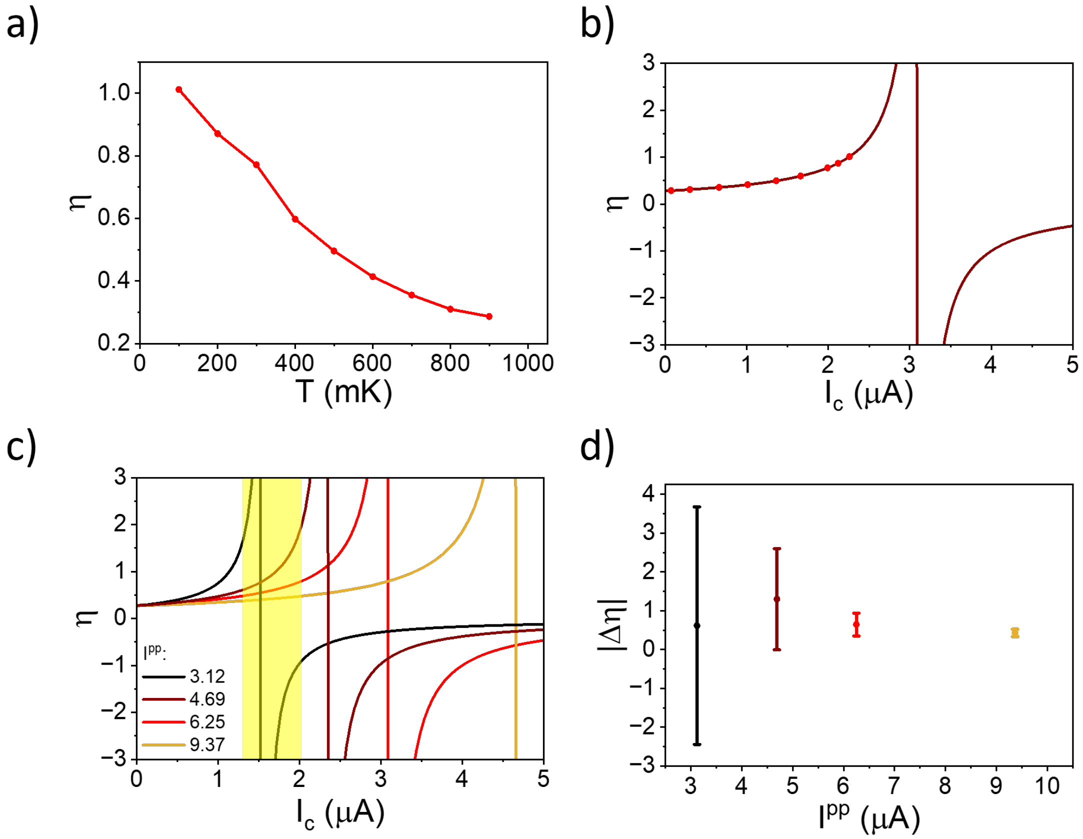

Figure 10: **Temperature dependence of the diode efficiency** a) $\eta(T)$ calculated with Eq. (1) and the experimental data of $I_c(T)$. b) $\eta(I_c)$ for a driving signal with $\eta_{ac} = 0.27$ and $I^{pp} = 6.25$ μA. The red dots are the value of η corresponding at the experimental data of $I_c(T)$. c) $\eta(I_c)$ for driving signals with different I^{pp}. The yellow region correspond to a fluctuation of $\Delta I_c \approx 0.6$ μA between 300 and 500 mK. d) Variation of diode efficiency in the yellow region of c) for the different driving signals.

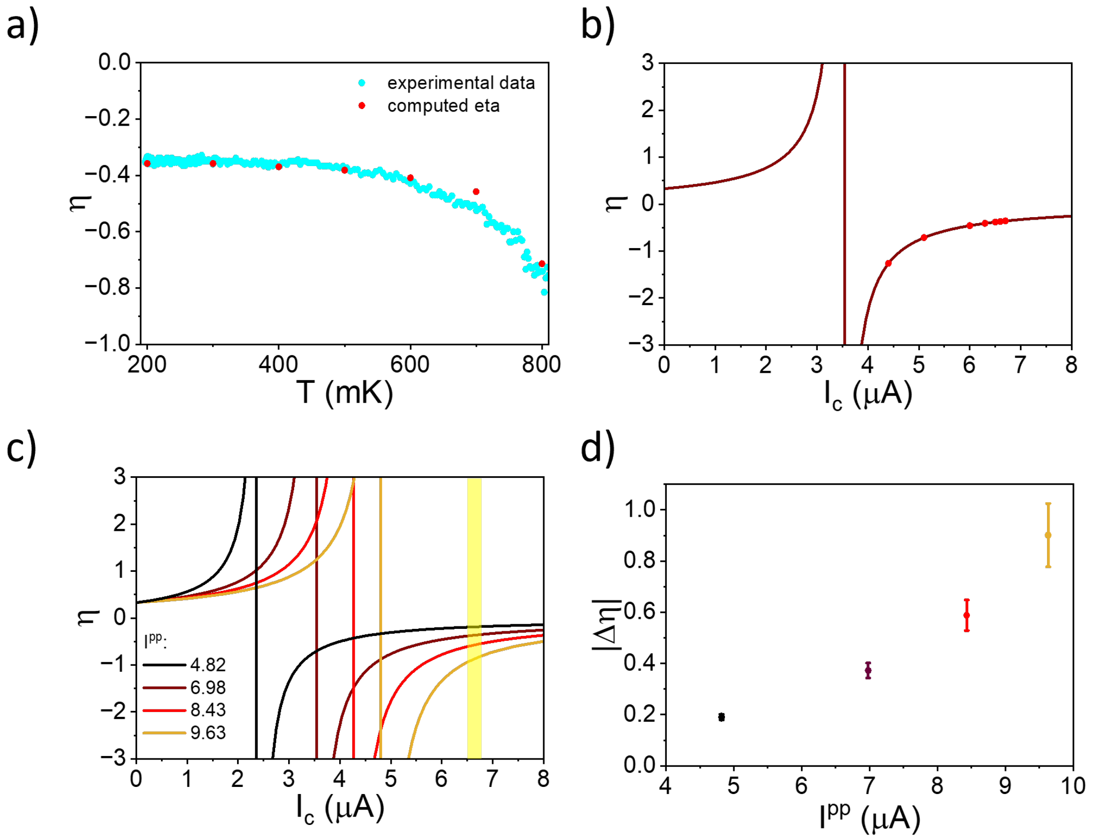

Figure 11: **Temperature dependence of the diode efficiency for Nb/Au/Nb JJ.** a) $\eta(T)$ measured (cyan) and calculated (red) with Eq. (1) and the experimental data of $I_c(T)$. b) $\eta(I_c)$ for a driving signal with $\eta_{ac} = 0.33$ and $I^{pp} = 8.43$ μA. The red dots are the value of η corresponding at the experimental data of $I_c(T)$. c) $\eta(I_c)$ for driving signals with different I^{pp}. The yellow region correspond to a fluctuation of $\Delta I_c \approx 0.2$ μA between 300 and 500 mK. d) Variation of diode efficiency in the yellow region of c) for the different driving signals.

References

(1) Paghi, A.; Trupiano, G.; De Simoni, G.; Arif, O.; Sorba, L.; Giazotto, F. InAs on Insulator: A New Platform for Cryogenic Hybrid Superconducting Electronics. *Advanced Functional Materials* **2025**, *35*, 2416957.

(2) Senesi, G.; Skibinska, K.; Paghi, A.; Shukla, G.; Giazotto, F.; Beltram, F.; Heun, S.; Sorba, L. Structural and Transport Properties of Thin InAs Layers Grown on InxAl1-xAs Metamorphic Buffers. *Nanomaterials* **2025**, *15*, 173.

(3) Paghi, A.; Borgongino, L.; Battisti, S.; Tortorella, S.; Trupiano, G.; Simoni, G. D.; Strambini, E.; Sorba, L.; Giazotto, F. Josephson Field Effect Transistors with InAs on Insulator and High Permittivity Gate Dielectrics. 2024; `http://arxiv.org/abs/2412.16221`.

(4) Paghi, A.; Borgongino, L.; Tortorella, S.; Simoni, G. D.; Strambini, E.; Sorba, L.; Giazotto, F. Supercurrent Multiplexing with Solid-State Integrated Hybrid Superconducting Electronics. 2024; `http://arxiv.org/abs/2410.11721`.

(5) Battisti, S.; De Simoni, G.; Braggio, A.; Paghi, A.; Sorba, L.; Giazotto, F. Extremely weak sub-kelvin electron–phonon coupling in InAs on Insulator. *Applied Physics Letters* **2024**, *125*, 202601.
